# Deep Learning of Fuzzy Weighted Multi-Resolution Depth Motion Maps with Spatial Feature Fusion for Action Recognition

**DOI:** 10.3390/jimaging5100082

**Published:** 2019-10-21

**Authors:** Mahmoud Al-Faris, John Chiverton, Yanyan Yang, David Ndzi

**Affiliations:** 1 School of Energy and Electronic Engineering, University of Portsmouth, Portsmouth PO1 3DJ, UK; mahmoud.al-faris1@myport.ac.uk; 2 School of Computing, University of Portsmouth, Portsmouth PO1 3DJ, UK; linda.yang@port.ac.uk; 3 School of Computing, Engineering and Physical Sciences, University of the West of Scotland, Paisley PA1 2BE, UK; david.ndzi@uws.ac.uk

**Keywords:** action recognition, transfer learning, multi-resolution, feature fusion

## Abstract

Human action recognition (HAR) is an important yet challenging task. This paper presents a novel method. First, fuzzy weight functions are used in computations of depth motion maps (DMMs). Multiple length motion information is also used. These features are referred to as fuzzy weighted multi-resolution DMMs (FWMDMMs). This formulation allows for various aspects of individual actions to be emphasized. It also helps to characterise the importance of the temporal dimension. This is important to help overcome, e.g., variations in time over which a single type of action might be performed. A deep convolutional neural network (CNN) motion model is created and trained to extract discriminative and compact features. Transfer learning is also used to extract spatial information from RGB and depth data using the AlexNet network. Different late fusion techniques are then investigated to fuse the deep motion model with the spatial network. The result is a spatial temporal HAR model. The developed approach is capable of recognising both human action and human–object interaction. Three public domain datasets are used to evaluate the proposed solution. The experimental results demonstrate the robustness of this approach compared with state-of-the art algorithms.

## 1. Introduction

Human action recognition (HAR) is a challenging field. This is due to a number of reasons. One important reason is the large variations in how actions are performed. It can also include variations in appearances of people, objects in their environment and the environment (scene). Deficiency in the availability of data resources, vague definitions of an action and drift in dynamic environments can all be sources of difficulty (see, e.g., [1]). This topic has been utilised in numerous applications such as surveillance based event detection, human–computer interaction and video retrieval [2].

Different conditions can also make HAR a difficult and challenging issue. There are issues such as occlusions, multiple viewpoints, action speed variations or even differences in illumination. Fortunately, depth cameras have enabled and provided a great push for HAR. They can provide both colour data as well as depth data. Depth is less sensitive to light intensity variances [3]. Depth is also more distinct than other derived appearance based features from, e.g., segmentation and detection [4]. Another aspect that can help make action recognition achieve better predictability is if data are taken as groups [1]. This is because groups of depth data for a single type of action can be more easily generalised to other instances.

In general, HAR can be classified into two categories: Hand crafted versus automatic learning methods. Hand-crafted feature based methods often consist of three stages. These are: Feature extraction, feature description and classification. Different kinds of hand-crafted features can be extracted from source sequences. Then, different descriptor techniques can be derived from extracted features. Finally, a classification process is usually employed to classify actions into different classes. Hand crafted features are useful because they enable some important aspects of the data to be emphasised. However, it is often difficult to make handcrafted features generally applicable. They can be sensitive to changes in a scene or even need to be re-designed for each application or scene [1]. These models are often not able to overcome challenging environments. On the other hand, automatically learned feature models can help overcome some of these shortcomings. Thus, one of the most important aims of this paper is to utilise both automatically and manually learned feature models for HAR.

**Figure 1:**
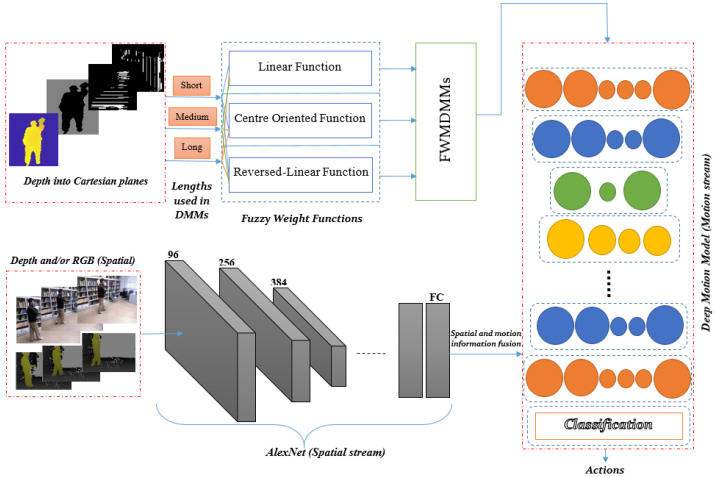
Framework of the proposed method.

In 2006, Hinton et al. proposed a solution for the training problem using a layer-wise training method based on deep learning [5]. Deep learning has been based on different techniques such as convolution [6], deep belief networks [5], auto-encoders [7] and recurrent neural networks [8]. These have all been used for learning of features, see, e.g., [9,10]. These kinds of approaches, in many cases, have been found to outperform hand-crafted features. Since then, much research has relied on deep learning techniques. It has been applied to various topics such as image classification [11], speech recognition [12] and object recognition [13]. In addition, many studies on HAR have used deep learning, either with colour image sequences or depth sequences [14,15,16]. However, most of these deep networks are based on either pre-extracted hand-crafted features or raw colour/depth sequences as inputs. This paper proposes a novel improvement on hand-crafted features based on fuzzy weighted multi-resolution depth motion maps (FWMDMMs). These help to characterise different important aspects of the depth motion data across multiple time resolutions. Then, deep learning is applied to automatically learn distinctive and compact features from the improved depth motion features. Important spatial information is learnt using the RGB and depth data using transfer learning. Finally, various fusion techniques are considered to suitably classify actions into appropriate classes. The proposed framework is illustrated in Figure 1.

### Main Contributions

In this paper, we propose a novel framework for learning of action models by combining together a deep and handcrafted hybrid feature model to get discriminative information. The key contribution of this paper is as follows:

- In order to learn information in the temporal dimension with diverse applicability, we develop a novel spatial temporal deep learning model. It includes a new method that improves traditional depth motion maps (DMMs) [17,18] called fuzzy weighted multi-resolution depth motion maps (FWMDMMs). The FWMDMM includes a number of different temporal model instances. These are used to help overcome the inherent variability in time associated for each individual action. Furthermore it can help overcome difficulties with self-occlusions and actions that might have similar types of movements.

The work differs from work in (e.g., [19,20]). For instance, for the DMM computation, overlapped segments were used in the calculation of a Multi-resolution DMM (MDMM). This means redundant information is included. For the work presented here, non-overlapped segments are used. This saves processing time for the MDMM calculation. Moreover, the weight functions of paper [19] are just incremental weights. For the work here, different weight functions are used. These enhance a number of different important aspects of an action sequence including incremental, decremented and middle weight functions.

In addition, the work presented here enhances the recognition system performance by including deep learning. This is used to process the DMM features and to provide more discriminative information for each action. Moreover, different streams of depth and appearance information are merged with the DMM information. This is performed with the use of different fusion techniques to produce the final spatio-temporal information.

The remainder of this paper is organised as follows: Section 2 reviews the related work. Section 3 presents the overall structure of the proposed framework and the detail of FWMDMM, fusion and deeper models. The experimental results and discussions are described in Section 4. Finally, Section 5 concludes the paper.

## 2. Related Work

HAR has been a popular area of research in the computer vision field. The performance of a HAR system depends on the quality of the features of a scene and of the actors themselves. Hence, many researchers have focused on designing superior scene descriptors. This has included using traditional approaches based on hand-crafted features to summarise and encapsulate local appearance and motion information effectively and efficiently. However, it has also included extension to include 3D information as well as the traditional appearance based RGB data.

Fortunately, cost effective depth sensors have been developed and have received increasing attention. Sensors such as Kinect [21] and Xtion [22] can provide depth information as an input. According to [23,24] an important advantage of depth based HAR, is in terms of the availability of 3D information. The 3D information of an object structure is useful in itself. However, it also helps to provide invariance to lighting and to help overcome other potential problems with, e.g., scale.

An effective HAR method, presented in [17], projected depth onto the three planes: Front, side and top views. Then, DMMs were generated encapsulating the motion information for entire video sequences for each DMM. In addition, Histograms of Oriented Gradients were computed from the resulting DMMs for use as descriptors. In [18], DMMs were used for HAR together with an $l_{2}$-regularised collaborative representation classifier with a distance-weighted Tikhonov matrix was also used. Chen et al. [25] calculated motion cues and local binary patterns (LBPs) from the DMMs. Two fusion levels were also considered including feature-fusion level and decision-fusion level. The DMM based results showed reasonable HAR performance.

Different levels of the same data sequence have been used with DMM computations to create a hierarchical DMM in [20]. A LBP based descriptor was used to characterise local rotation invariant texture information. Then a Fisher kernel was employed to create patch descriptors. These were then fed into a kernel-based extreme learning machine classifier. A similar approach was followed by [26]. A histogram of oriented gradients (HOGs) descriptor was used along with kernel entropy component analysis for dimensionality reduction. Finally a linear support vector machine was used in the classification. For both hierarchical DMM based approaches, the results demonstrated a significant performance improvement. DMMs can express the variation of a subject’s motions during the performance of an action. However, difficulties can potentially arise between actions that have the same type of movements but over different temporal periods. To tackle this issue, fuzzy weighted multi-resolution depth motion maps are proposed here in this work, explained in the next section.

Some other researchers have used skeleton joint information for depth based HAR such as [27]. The skeleton joint information aided in finding the relationship between different body parts. A combination of various interest point detectors were used in [28]. These formed different space time interest points (STIPs) features. The experiments demonstrated that the recognition rate could be improved by combining skeleton joint information and spatio-temporal features. However, it is dependent on deriving an accurate skeleton representation. Other methods have been proposed to represent depth sequence information without the need to derive the skeleton information. This has included learning an actionlet ensemble [29], spatio-temporal depth cuboid features [30] and super normal vectors [31].

Deep learning has successfully been used for HAR [32,33,34]. Deep learning models have the ability to learn features in a hierarchical way starting from low level features reaching to high level ones. convolution neural networks (CNNs), were proposed in [6]. CNNs use trainable filters and local neighbourhood pooling processes to obtain a hierarchy of complex features. CNNs can be made invariant to variations in pose, lighting and surrounding clutter [35]. In addition, CNNs can achieve great performance on visual field tasks when trained with proper regularisation [36,37]. convolutional neural network models have been used to build deep learning based HAR systems such as [38]. Video based spatial and temporal information was learned. A deep two-stream model was constructed based on transfer learning using a modified RestNets-101. A stream is a series of layers trained on a set of features. RGB and a volume of stacked optical flow data were taken as inputs for the spatial and temporal network streams, respectively. The proposed strategy was able to achieve competitive results for HAR.

For a fixed orientation, a spatio-temporal convolutional network based HAR was proposed in [39]. This used spatio-temporal features. Moreover, in [40], a HAR system was proposed based on a two stream deep CNN model. The two streams consisted of a spatial stream which learned appearance features and a temporal stream which learned motion information. The motion information was based on stacked optical flow frames. An extension of this two stream network approach was proposed in [41] using dense trajectories. This resulted in more effective learning of motion information. A fusion of two stream networks was proposed in [42] by applying several combinations between them. This helped to take further advantage of the spatio-temporal features.

A general residual network architecture for HAR was presented in [43]. Here cross-stream residual connections in the form of multiplicative interaction between appearance and motion streams were used. The motion information was exploited using stacked inputs of horizontal and vertical optical flow. A fusion study was presented in [44] for HAR. Two streams of the pre-trained visual geometry group (VGG) network model were used to compute spatio-temporal information combining RGB and stacked optical flow data. Various fusion mechanisms at different positions of the two streams were evaluated to determine the best possible recognition performance.

All of the above approaches suffer from a shortage of long term temporal information. For example, the number of frames used in the optical flow stacking ranged between 7 and 15 frames. For example, 7, 10 and 15 frames were used by [35,42,45], respectively. Often people will perform the same action over different periods of time depending on many factors and particularly for different people. Consequently, multi-resolution hand-crafted features computed over different durations of time are used here in this work. This helps to avoid this problem. Furthermore, different weight phases are applied using a fuzzy algorithm in the computation process of the DMMs. Thus, enabling adaptation to different aspects of an action.

We take advantage of a deep learning method to learn discriminative features from both RGB and depth sequences. At the same time, we exploit some important aspects a priori by developing hand-crafted features in a deep motion model. These hand-crafted are referred to here as FWMDMMs. The FWMDMMs are extracted from depth sequence data and learned as part of the motion information. Two streams are considered here, including the deep motion model and a pre-trained AlexNet model to extract the motion and spatial features, respectively. Moreover, different fusion techniques are evaluated. These are used to merge the spatial and motion information to find the best way approach for HAR models proposed here.

## 3. Construction of Fuzzy Weighted Multi-Resolutions Depth Motion Map

### 3.1. Depth Motion Maps (DMMs)

Both RGB and depth for each frame are exploited in the framework proposed here. In addition, multi-resolution shape and motion information are also used here in the form of a modified DMM formulation. The basic DMM, (as used in, e.g., [17,18,46]), includes projecting each depth frame onto three orthogonal Cartesian planes. The foreground is specified by a bounding box as a region of interest and then normalised to a particular size.

As a result, each depth frame will generate three 2D planes or maps xy, xz, yz indicating front, side and top views. The motion energy of each single map can be obtained by computing and thresholding the difference between two consecutive maps. This provides a superior clue for HAR by specifying motion regions and showing where the motion occurs in each temporal template.

The motion energy can then be stacked through a specific interval or through the entire sequence. This generates a DMM, $\Gamma_{v}$ for each projection view,

(1) $$\Gamma_{v}=\sum_{t=1}^{N-1}\left|m_{v}^{t+1}-m_{v}^{t}\right|$$

where v∈ { xy, yz, xz } indicates the projection view; $m_{v}^{t}$ is the projected map of frame *t* under projection view *v*; *N* is the number of frames that indicates the length of the interval.

In general, DMMs are represented on each orthogonal Cartesian plane by combining projected maps of an entire depth sequence. Hence, important information of body shape and motion are emphasised.

### 3.2. Multi-Resolution Depth Motion Maps (MDMMs)

Traditional DMMs are formulated on 2D planes as described above by combining projected motion maps of an entire depth sequence. This formulation does not consider the higher order temporal links between frames of depth sequences. An enhancement is therefore applied here to improve on the traditional DMMs.

Mostly, a fixed number of frames have been used by other researchers or even the entire number of frames of an action sequence video. However, a length of an action is not known in advance. In addition, an action can be performed at different speeds by different people. Hence, MDMMs are used here to cover different temporal intervals and rates of an action.

**Figure 2:**
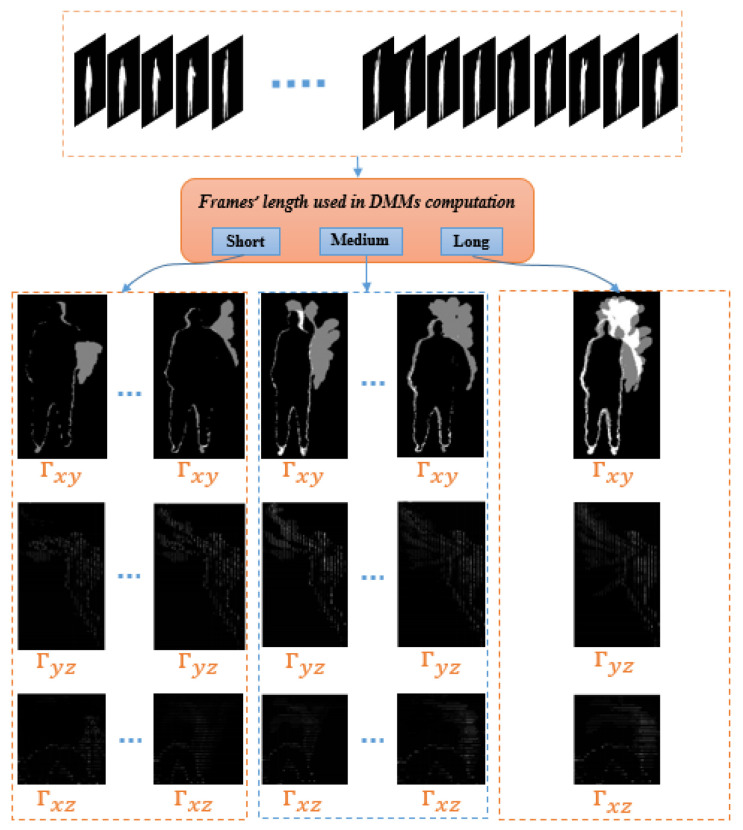
Computation procedure of the multi-resolution depth motion maps (MDMMs).

In our work, the depth sequence is split into three different groups where each has a different time interval. This means, various values of the threshold, τ, formulated to generate MDMMs for the same action (depth sequence). As τ∈$N^{+}$ in traditional DMMs, this can be improved by τ∈$\left\{g_{1},g_{2},g_{3}\right\}$ where $g_{i}$∈$N^{+}$. This extension enables different temporal windows to properly cover an action’s motion regardless of whether it carries important information over a short or long duration. Each of these three durations produce a different DMM. The values of τ are selected to cover short, intermediate and long durations. For long, this would typically correspond to an entire depth sequence for the various video sequences considered here. Figure 2 illustrates the computational procedure for MDMMs.

These MDMMs for each depth sequence can be calculated with:

(2) $$\Gamma_{v,\tau ,t}=\sum_{t^{{}^{\prime }}=t}^{t+\tau -1}\left|m_{v}^{t^{{}^{\prime }}+1}-m_{v}^{t^{\prime }}\right|\;\forall \;\tau$$

where $v\in \left\{xy,yz,xz\right\}$, τ∈λ and, e.g., $\lambda =\left\{5,10,All\right\}$ are the various lengths of depth sequence used to obtain a MDMM for each single frame.

Then, a single MDMM is created again via concatenation, but now across all time resolution windows τ∈λ after processing, i.e., $\Gamma ={||}_{\tau \in \lambda }\Gamma_{\tau }$, where || represents concatenation.

### 3.3. Fuzzy Weighted Multi-Resolution DMMs (FWMDMMs)

In order to identify significant motion, we take into consideration the motion history image based method. A linear weighting as a function of time is used to find historical motion information with high weight due to recent motion. Hence, after weight allocation for each frame, the recent moving pixels which have most recently shown some motion result in greater intensities. By proceeding with this approach, a fuzzy algorithm is used here to give each DMM various weights to assign dynamically ranging importance to the motion information.

**Figure 3:**
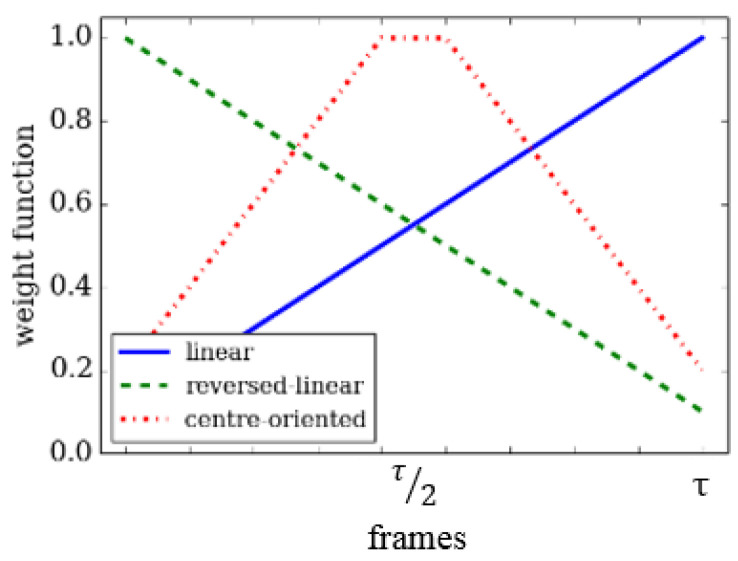
Fuzzy weight functions based weighted depth motion maps (DMMs).

**Figure 4:**
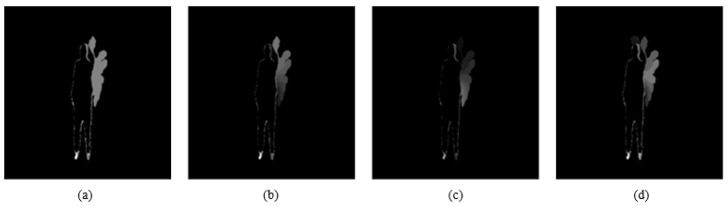
$DMM_{xy}$ based on different aspects of fuzzy weight functions: (**a**) Original DMM uniform weight, (**b**) linear weight function, (**c**) Reversed linear weight and (**d**) centre-oriented weight function.

Let $w_{t}$ be the linear weight that is given to frame *t* with values in the range between $w_{t}\;\in \;\left[0,1\right]$. This can be improved for DMMs by replacing the $w_{t}$ with three fuzzy weight functions. Each has the same range based weighting approach. This helps to emphasise salient aspects in time of an action sequence. The three weight functions provide three different weight formulations for frames in the same DMM template. The result is three different DMM representations. These fuzzy functions can assign importance to the motion information in frame *t* using three linearly varying functions. The functions are: linear function, reversed linear function and central-oriented function. Figure 3 and Figure 4 illustrate the fuzzy weight functions and the resulting effects on the DMMs ($DMM_{xy}$ is considered in the figure).

- Linear varying function
  A linear fuzzy membership function that increases the importance of motion information linearly with time for frame *t* in the DMM template computation. This can be formulated as:
  (3) $$F_{1}\left(t\right)=\frac{t}{\tau }\;\mathrm{where}\;t\in \left[1,\tau \right].$$
  where τ∈λ.
- Reversed linear function
  Reversed linear based membership function performs the reverse of the linear membership function. It decreases the importance assigned to motion information with time in frame *t* in the DMM template computation. This can be formulated as:
  (4) $$F_{2}\left(t\right)=1-\left(\frac{t-1}{\tau }\right)\;\mathrm{where}\;t\in \left[1,\tau \right].$$
- Central oriented function
  For the case of the centre based membership function, high importance is assigned to motion information in the middle frame of the DMM templates and decreases to the two sides. This membership can be formulated as:
  (5) $$F_{3}\left(t\right)=\left\{\begin{array}{c} \frac{2t}{\tau }\;\;\;\;\;for\;\;\;\;\;1<t\leq \;\frac{\tau }{2};\\ 2\left(1-\frac{t-1}{\tau }\right)\;\;\;for\;\;\frac{\tau }{2}<t\leq \tau . \end{array}\right.$$

The weighting function is combined with the MDMM like so:

(6) $$\Gamma_{v,\tau ,t,j}=\sum_{t^{{}^{\prime }}=t}^{t+\tau -1}\left|m_{v}^{t^{{}^{\prime }}+1}-m_{v}^{t^{{}^{\prime }}}\right|F_{j}\left(t^{{}^{\prime }}\right).$$

The final version of improved DMMs are generated using three temporal templates based on the intervals $\left\{\mathrm{short},\;\mathrm{medium},\;\mathrm{long}\right\}$. These are weighted with fuzzy functions to produce fuzzy weighted DMM templates. Concatenation is used after processing to produce a FWMDMM, based on a particular weighting function.

## 4. Deep Convolutional Neural Networks

Convolutional neural networks (CNNs) are a type of multi-layer Perceptrons that can be considered to follow the same principles as the visual mechanisms in organism. At the most basic level there are cells combined together in the form of various complexities assigned to sub-regions of a visual scene. A similar although considerably simplified kind of processing can be achieved by using convolutional filters or CNNs over a given data.

CNNs can automatically achieve feature extraction directly from input data. This can help solve the exhaustive search of hand-crafted methods. Furthermore, CNN operations include the local receptive field, shared weights and pooling. These processes are adopted to attain shift, scale and distortion invariance that can improve recognition accuracy.

### 4.1. Transfer Learning of Spatial Information

**Figure 5:**
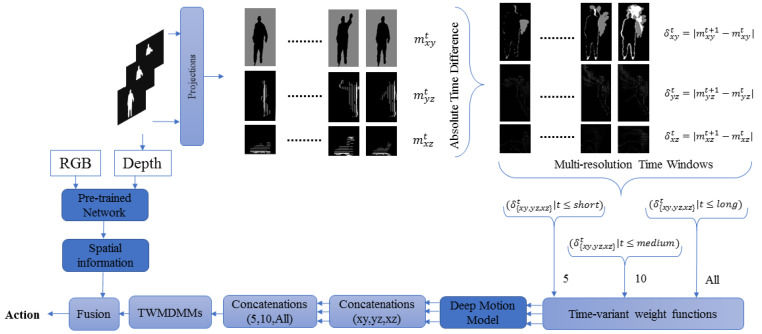
Recognition system stages using FWMDMM hand-crafted features and spatial information via deep motion model and pre-trained AlexNet network.

A pre-trained AlexNet model proposed in [47] containing eight pre-trained layers is used here for initialisation. Two parallel AlexNet networks are used here to compute features of the RGB and depth information. In addition, extra CNNs are used for the motion model to fuse and learn the information of the two parallel networks and DMM hand-crafted features. Figure 5 shows all stages of the recognition system proposed here. While there are many better options of pre-trained networks, AlexNet was used in transfer learning due to its moderate trainable parameters that can meet our processing limitations. AlexNet has been widely used for HAR in many research studies due to its reasonable trainable parameters in comparison to other pre-trained network models see, e.g., [48].

Network architecture is very important because it plays a significant role in the performance of the deep learning model. Common deep network architectures usually have alternating convolutional layers and auxiliary layers, e.g., pooling, rectified or dropout layers and terminated by a few fully connected (FC) layers.

Originally in [47], the output of the third fully connected layer was delivered by using a 1000 softmax functions which assigns the distribution over 1000 class labels. In our case, the last fully connected layer is delivered in a different way using softmax functions based on the number of existing class labels of the validation datasets.

The input image of the first convolution layer is filtered with 96 kernels of size 11 × 11 × 3 and a stride of 4 pixels. Next, the output of the first convolution layer is taken as input to the second convolution layer and filtered with 256 kernels of size 5 × 5 × 48. The following layers are connected one to another with 384 kernels of size 3 × 3 × 256, 384 kernels of size 3 × 3 × 192 and 256 kernels of size 3 × 3 × 192 in terms of the third, fourth and fifth convolutional layers, respectively.

### 4.2. Fusing the Spatial Networks

For more reliable recognition, it is preferable to utilise diverse information sources combined using fusion to achieve better performance. This can be done using either concatenation of features or via an average of several decision scores [42]. Fusion can be utilised to combine any given set of deep networks. An easier way to combine multiple networks is to add an extra fully connected layer to combine outputs of the networks. The advantage of using different features at the same time is to help improve the recognition model and hence recognition performance.

In our work, we propose multiple different fusion techniques at different positions of the spatial two-stream networks. Information fusion is implemented partially between the RGB and depth stream networks combining multiple layers of two trained parallel networks including several CNN architectures such as early, middle and late fusion. This helps to find the best position and technique for RGB and depth fusion that can optimise the recognition rate. In addition, hand-crafted features are exploited in the deep motion model using improved DMMs as an auxiliary source of features that represent motion information of an action. Fusion position via motion model is specified based on the best recognition result of the fused spatial two-stream networks. Then, fused spatial information is utilised with the motion information in the deep motion model using the same fusion position. By adding hand-crafted features, our approach can incorporate two explicitly different types of features such as spatial and temporal information into the classification process.

In this section we consider different architectures for fusing the spatial two-stream networks for RGB and depth. Spatial fusion can be achieved between the two networks when the networks have the same spatial resolution at the fused layers by adding, multiplying or concatenating layers from one network to another. Later, the subsequent layers can suitably learn the correspondence between these channels to recognise the action.

A number of fusion techniques that are used between the two spatial networks are described here. Moreover, the consequences of each technique are highlighted in the experiments section. Let $f:R^{H\times W\times D}\times R^{H\times W\times D}\to R^{H\times W\times D}$ be a fusion function which fuses two feature maps $x_{t}^{a}\;\in \;R^{H\times W\times D}$ and $x_{t}^{b}\;\in \;R^{H\times W\times D}$ that belong to two different networks to produce an output $y_{t}\;\in \;R^{H\times W\times D}$, where *H*, *W* and *D* are the height, width and number of channels of the feature maps, respectively. The number of feature maps are based on the specific architecture of the network (in our case, there are 16×16=256 for convolutional layer 5). Function *f* can be employed at various stages in the networks to achieve early, mid or late fusion.

- Sum fusion is employed to compute the sum of the elements of two feature maps where each has the same spatial location and feature channels. Let *d* be the number of feature channels, then:
  (7) $$y_{i,j,d}^{\mathrm{sum}}=x_{i,j,d}^{a}+x_{i,j,d}^{b},$$
  where 1<i≤H, 1<j≤W, 1<d≤D.
- Multiplicative fusion computes the multiplication of the two feature maps at each pixel location:
  (8) $$y_{i,j,d}^{\mathrm{multip}.}=x_{i,j,d}^{a}\;\cdot \;x_{i,j,d}^{b}.$$
- Concatenation fusion concatenates the two feature maps at the same spatial location and cross feature channels:
  (9) $$y_{i,j,d}^{\mathrm{cat}}=x_{i,j,d}^{a}\left|\right|x_{i,j,d}^{b}.$$

The proposed method is implemented with the various fusion techniques to fuse the RGB and depth spatial networks. These are applied at different positions between the spatial networks such as at the convolutional, max-pooling or fully connected layers. The output of the spatial fusion can be used to train a supervised classifier (KNN) to find the best position of fusion. Then, spatial (fused RGB and depth) and motion information are fused. Finally this is used to train a classification layer trained with standard back-propagation and stochastic gradient descent based algorithms.

### 4.3. Deep Motion Model

**Figure 6:**
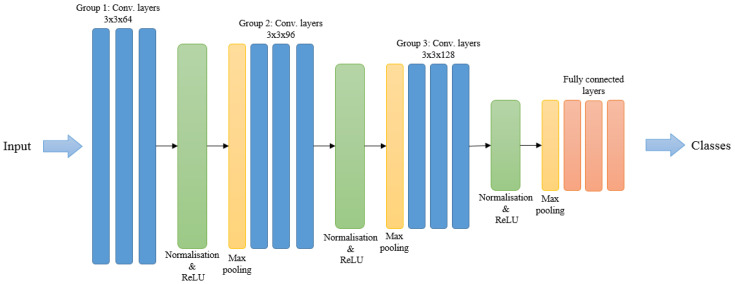
Architecture of the deep motion network.

After spatial fusion stages, we propose to go deeper to represent the temporal information in a proper way to help utilise the highly discriminative motion features. Our deeper motion model consists of a CNN based architecture that employs multiple distinct convolution operations to help identify discriminative features. It contains eleven learned layers including nine convolutional layers and two fully connected layers. We have designed various CNN architectures based on the deep temporal information but the results show that this architecture achieves the best performance. A brief description of the network architecture is shown in Figure 6.

The convolutional layers constitute three groups in the network. Each has three convolutional layers, separated by normalisation, ReLu and max-pooling layers. The specifications of the later three layers are the same after each single group in the network. The specification of the convolutional layers are varied according to group stage. The hand-crafted (FWMDMMs) information proposed in Section 3.1 forms the input to the motion model. This starts at the first group of convolutional layers and filtered with 64 kernels of size 3 × 3 each. Next, the output of the third convolutional layer in the first group is fed into the normalisation and max-pooling layers. These have pool size [2, 2] and stride [2, 2].

The output is then fed to the second group of three convolutional layers. These filter with 96 kernels of size 3 × 3 each. The output is then normalised and down-sampled again by the normalisation and max-pooling layers with the same specifications.

The final convolutional group consists of three convolutional layers. These filter the output of the previous layer with 128 kernels of size 3 × 3 each. The normalisation and down-sampling implemented layers then feed the information to the fully connected layers. These are separated by a dropout layer to give a significant boost to the performance of our model.

## 5. Implementation

The parameters of the fuzzy weight functions in Equations (3)–(5) are wholly dependent on the lengths of the weighted sequence τ as utilised by Equation (6). The length of the weighted sequence is varied as part of the multi-resolution approach, i.e., τ∈{5,10,All}. This removes dependence on a single window length and consequently the underlying parameter settings too.

The system was implemented using Matlab. An NVidia 2GB Quadro Pro GPU was used to speed up the implementation process and to facilitate the deep learning techniques.

The AlexNet model was fine-tuned using stochastic gradient descent with batch size equal to 100, momentum 0.9 and weight decay 0.0005. The update rule for weight *w* is $w_{i+1}:=w_{i}+v_{i+1}$ for iteration *i* with momentum variable:

(10) $$v_{i+1}:=0.9\cdot v_{i}-0.0005\cdot \varepsilon \cdot w_{i}-\varepsilon .{\langle \frac{\partial L}{\partial w}{|}_{w_{i}}\rangle }_{D_{i}}$$

where ε is the learning rate and ${\langle \frac{\partial L}{\partial w}{|}_{w_{i}}\rangle }_{D_{i}}$ is the average over batch *i* of $D_{i}$ of the derivative of the objective with respect to *w*, evaluated at $w_{i}$.

The biases in AlexNet are initialised with constant 1 in the second, fourth and fifth convolution layers in addition to the fully connected layers. The biases are set to constant 0 for the remaining layers. The same learning rate was used for the early layers and increased for the latter layers in order to accelerate the training. The learning rate was initialised at 0.001 [47].

The time complexity of the DMM stream is two frames per second in the training section. While the testing time is 11 frames per second. It is difficult to make a like for like comparison with other state-of-the-art methods due to differences in hardware and software. However, the proposed system is able to run in real-time or close to real-time with relatively modest compute hardware.

## 6. Experimental Results and Discussion

The proposed approach was evaluated on the Northwestern-UCLA multi-view action 3D dataset [49], MSR 3D daily action dataset [50] and MSR 3D actions dataset [51]. These datasets contain RGB-D videos captured using Microsoft Kinect based depth sensors. In this work, we compute the FWMDMM temporal information for each observation in these datasets from the depth sequences. To obtain additional discriminative information of an action we do exploit RGB and depth sequences captured by Kinect sensors. In each, features are extracted from both RGB and depth information using the pre-trained AlexNet network after fine-tuning the network. Various fusion techniques are used to fuse both sources of information at different positions of the networks to show their effects on the recognition rate. Furthermore, the CNN motion model is used to process the motion information (FWMDMMs). This is then fused with spatial information (RGB and depth). The result is highly discriminative features combining both spatial (RGB and depth) and motion (FWMDMM) information. Further experimental details and results for these datasets are given in the following sub-sections.

### 6.1. Northwestern-UCLA Multi-View Action 3D Dataset

**Figure 7:**
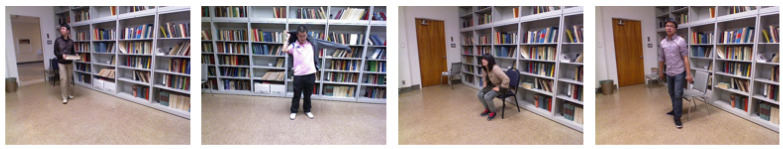
Samples of the NUCLA dataset.

Northwestern-UCLA (NUCLA) multi-view 3D event dataset has three Kinect cameras used to capture RGB, depth and human skeleton data simultaneously. This dataset includes 10 different action categories including: pick up with one hand, pick up with two hands, drop trash, walk around, sit down, stand up, donning, doffing, throw, carry. Each action is performed by 10 actors. Figure 7 shows some example frames of this dataset. In addition, this dataset consists of a variety of viewpoints.

We evaluate our proposed method with two different training and testing protocols for this dataset:

- Cross-subject training scenario: In this setting we use the data of nine subjects as training data and leave the data of the remaining subject as test data. This is useful to show the performance of the recognition system across subjects. Furthermore, this is a standard criteria for comparison with the state-of-the-art.
- Cross-view training scenario: As this dataset contains three view cameras, we use the data of two cameras as training data and leave the remaining camera as test data. This kind of setting is used to demonstrate the ability of the recognition system against different views and to get another standard criterion to compare with the state-of-the-art.

These settings give the opportunity to evaluate the robustness of the proposed method to variations in different subjects and different views. The proposed method achieves an interesting set of results with the complete system demonstrating state-of-the-art performance. However, first let us examine the performance of the individual subsystems. In the beginning the classification performance using the pre-trained AlexNet network is investigated for the aforementioned scenarios. Table 1 includes the results of the spatial based AlexNet implementation in terms of depth and RGB information.

Then, we fuse both RGB and depth parallel streams to constitute the spatial information. In this step we employ different fusion techniques at different positions between the two networks. We choose four positions to implement the fusion: Conv5, Max-pooling3, FC7 and FC8 layers to find the most suitable position for fusion. The fusion output is fed to a supervised classifier. In our case the KNN classifier is chosen for recognising an action. Table 2 includes the recognition results using spatial information for the different fusion positions.

As can be seen from Table 2, the results improve gradually whenever the fusion occurs at deeper levels. This is possibly due to the improvement in the overall set of features being extracted at each layer. Taking this result, an additional fusion is used to fuse spatial (RGB and depth) and motion (FWMDMMs) streams at a fully connected (FC) layer. This was done to find out how the spatial information (RGB and/or depth) can affect the recognition system. This is important because the hand-crafted FWMDMMs features are merged with spatial information in the motion model. Table 3 includes the performance results of the recognition of the deep motion model. Included are the with and without fusion results for the spatial model. It also includes a comparison between traditional DMMs and improved MDMMs. As can be seen from the results in Table 3, the inclusion of spatial information significantly improve the results; whilst the MDMM combined with the spatial information is the best overall. Table 4 includes the performance results with the FWMDMMs as proposed here. It includes different fuzzy weights in the DMM computation. These are then fused at the fully connected (FC) layer with the spatial information using the different fusion techniques.

It is clear from Table 3 that the use of MDMMs is significantly better in comparison to DMMs for HAR because of their ability to cover different periods of an action. This is most likely due to an improved ability to cope with inherent variability for a performed action. In addition, it is obvious that using spatial information with the motion information can improve the recognition rate of the system.

Many experiments have been undertaken here to find the most suitable information for HAR. These demonstrate that the spatial information (depth and RGB) have a significant effect on the recognition system. This appears to be particularly true when the spatial information is combined with the motion information. It can be understood because FWMDMMs can cover a wide range of an action simultaneously emphasising the temporally important parts of each action. The highest performance is achieved using FWMDMMs, as proposed here in this work. This is for different temporal weighting concatenated with the spatial information. It can be seen in Table 4 that the FWMDMM based approach achieves some enhancement in the performance, reaching 98.89%. This method is compared with some state-of-the-art approaches as seen in Table 5.

The results show that virtual view [52] and Hanklet [53] methods are limited in their performance. This reflects the challenges of the NUCLA dataset (e.g., noise, cluttered backgrounds and various viewpoints). To help mitigate against these challenges, MST-AOG was proposed in [49] and achieved 81.60%. Our method achieves a significant improvement of 18% over MST-AOG and some comparable performance for the cross-view setting. This is due to the big challenge in a cross-view setting. In a cross-view setting, a scene in one view is different from the same scene in another view. The distribution of the extracted features varies from one view to another. This is because of the difference between the motion and appearance cues of an action across multiple views. The system was trained here on a single view and testing was performed with the remaining views. A confusion matrix of the proposed method is shown in Table 6.

### 6.2. MSR Action 3D Dataset

**Figure 8:**
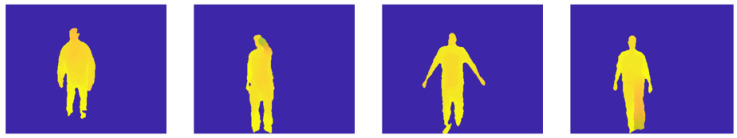
Samples of the Microsoft Research (MSR) 3D action dataset [51].

The Microsoft Research (MSR) action 3D dataset [51] is an action dataset consisting of depth sequences with 20 actions: High arm wave, horizontal arm wave, hammer, hand catch, forward punch, high throw, draw cross, draw tick, draw circle, hand clap, two hand wave, side-boxing, bend, forward kick, side kick, jogging, tennis serve, golf swing, pickup and throw. Each action is performed three times each by ten subjects. A single point of view is used where the subjects were facing the camera while performing the actions. Samples of actions of MSR action 3D dataset are shown in Figure 8. The dataset has been split into three groups based on complexity in most of literature studies such as [17,18,51,62].

As the dataset is split into three subsets: AS1, AS2 and AS3 each has its own actions based on the complexity as included in Table 7, all validation schemes are used with the three subsets respectively.

Three evaluation schemes are considered in the literature (see, e.g., [63]) in terms of MSR action 3D dataset: 1/3, 2/3 and cross-subject.

- The 1/3 evaluation scheme: 1/3 of the instances are used as training samples and the reminder as testing samples. The 1/3 scheme splits the dataset using the first repetition of each action performed by each subject as training and the rest for testing.
- The 2/3 evaluation scheme: 2/3 of the instances are used as training samples and use the remainder as testing samples. The 2/3 scheme splits the dataset into training samples using two repetitions of each action performed by each subject and testing using the rest of the data.
- The cross-subjects evaluation scheme: Half of the subjects are used as training samples, the other half used as testing samples. Any half of the subjects can be used for testing, e.g., 2, 4, 6, 8 and 10; and the rest for training, i.e., 1, 3, 5, 7 and 9 (as used here).

Each subset has eight actions that can used to evaluate the proposed method. This is in terms of proportions 1/3 and 2/3. Moreover, cross-subject validation schemes are used to assess the performance of the proposed method against different training settings. This can include shortage of training samples, many training samples and variations between subjects.

The same procedures for the above experiments were used for the MSR 3D actions dataset. The pre-trained AlexNet network classification was trained based on the depth sequence data only for all evaluation schemes. Table 8 includes the results of the AlexNet implementation in terms of depth sequences.

As this dataset has no RGB data, only depth sequences are used. The depth data is the only spatial information employed in the fusion with the deep motion model. Table 9 includes the results of the deep motion model using traditional and improved DMM features. These are combined at the FC layer with spatial information (depth sequences).

The results in Table 9 enable a comparison with traditional DMMs and MDMMs. The results appear to show that MDMMs merged with spatial information offer some improvements. Based on that observation, different temporal fuzzy weights were then considered. These included linear, reversed linear and central-oriented based weighted MDMMs. These are used in our deep motion model. These were fused at the fully connected (FC1) layer with the spatial information. Table 10 includes the performance of the proposed method. It illustrates the improvements for different fusion computation approaches. Concatenation, multiplication and addition fusion calculations are compared.

**Figure 9:**
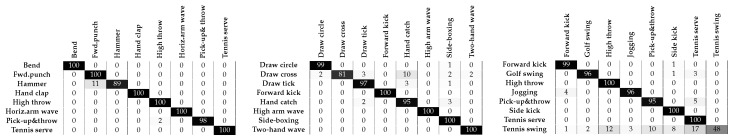
Confusion matrix of spatial information and central-oriented fuzzy weighted multi-resolution DMMs (FWMDMMs) based method, using CS validation scheme in terms of AS1 (**left**), AS2 (**middle**) and AS3 (**right**) subsets of MSR 3D action dataset.

The concatenation fusion approach appears to provide the best performance for all cases. The centre based approach appears to perform reasonably and consistently well for the majority of the cases, particularly for the concatenate fusion approach. Figure 9 shows the confusion matrices of the recognition system. This is for the FWMDMMs under the above regulations in terms of the MSR action 3D dataset.

A comparison between the proposed method and the state-of-the-art approaches for HAR is presented in Table 11. These results are for the MSR action 3D dataset. Our proposed method appears to outperform the state-of-the-art approaches for the majority of cases. In others, it appears to achieve at least comparable performance. Some of these other methods are DMM based, such as [17,25]. In these cases, our method achieves greater recognition rates in the range of 1–6%. This could possibly suggest that FWMDMM and spatial information based features can help to provide more powerful discrimination. Our approach also utilises multiple hierarchical features that cover various periods of an action. In addition, our deep recognition model uses a diverse range of layers. These things combined appear to help to improve the chances to obtain the most accurate information. Many of the better performing methods (in terms of cross-view) typically rely on the use of skeleton data. This can be considered a potential disadvantage. This is due to the a priori information that skeleton extraction algorithms typically rely. This can be seen with the final set of results presented in Section 6.3. The methods that put too much reliance on skeleton data are not able to perform as well when errors occur with the inferred skeleton information.

### 6.3. MSR Daily Activity 3D Dataset

**Figure 10:**
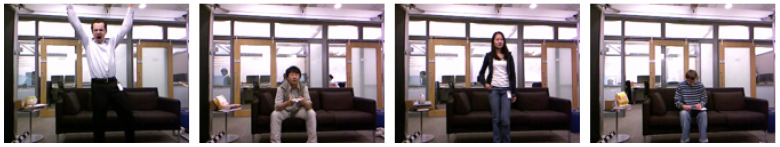
Samples of the MSR daily action dataset.

The MSR Daily activity 3D dataset is quite a challenging dataset. This is because of a high level of intra-class variation. Furthermore, some of the actions are based on object-interaction. This is where the subject is interacting with an object when performing an action. The dataset has been captured by a Kinect sensor at Microsoft Research Redmond. It consists of depth and RGB sequences. It includes 16 actions: Drink, eat, read book, call cellphone, write on a paper, use laptop, use vacuum cleaner, cheer up, sit still, toss paper, play game, lay down on sofa, walk, play guitar, stand up, sit down. Performed by 10 subjects each subject performs an action twice in two different poses (standing and sitting). Samples of this data set can be seen in Figure 10.

Different evaluation schemes have been considered in the literature in terms of MSR daily activity 3D dataset. Here, similar to [19,30], the sequences in which the subjects were almost still are not considered. As a result, ten types of action are taken into account in our experiments including: Drink, eat, call cellphone, use vacuum cleaner, cheer up, toss paper, lay down on sofa, walking, stand up and sit down. A cross-subject validation was performed with subjects 1, 3, 5, 7, 9 for training and subjects 2, 4, 6, 8, 10 for testing.

The same procedures for the previous experiments are implemented regarding MSR daily activity 3D dataset. The pre-trained AlexNet network classification is implemented based on the depth and RGB sequences under the evaluation scheme. In addition, a fusion between RGB and depth is considered and fed to a supervised classifier. In our case the KNN classifier is used to recognise an action to find the most suitable position of fusion. Table 12 includes the results of the AlexNet implementation before a fusion and KNN classification post fusion.

The RGB and depth sequences which form the spatial information are then fused with the deep motion model. Table 13 includes the results of the deep motion model using traditional and improved DMM features concatenated with spatial information.

It is worth noting in Table 13 that the recognition rate improved after fusion between the spatial and motion information, outperforming the traditional DMMs and MDMMs based methods. Taking this into consideration, different fuzzy weights (linear, reverse linear and central-oriented) based FWMDMMs in the deep motion model were compared. They were then fused at the fully connected (FC1) layer with spatial information by various fusion techniques. Table 14 includes the performance of the proposed method based on these different fuzzy weight functions for the MSR daily activity 3D dataset.

The results in Table 14 appear to show that the centre-oriented fuzzy weight function and the concatenation based fusion approach provide the best overall result. To assess the efficiency of the proposed method, a comparison between the state-of-the-art approaches and the work proposed here is presented in Table 15. This is in terms of the MSR daily activity 3D dataset.

In Table 15, it can be seen that limited accuracy was previously achieved by LOP [29] and ROP [75] based approaches. DSTIP+DCSF [30] was designed to overcome some of the limitations posed by the work of [75], achieving a recognition rate of 83.60%. Actionlet Ensemble in [29] achieved an 86% recognition rate using a combination of depth and skeleton data. A recent method in [19] indicated the difficulty of skeleton data capturing process. It suggested the use of depth sequences based on temporal DMMs and Fisher kernel representation. It achieved a relatively competitive result of 89%. Our method achieves an improvement of 3.88% using FWMDMM with spatial information. The confusion matrix in terms of MSR daily activity 3D dataset is shown in Table 16.

**Figure 11:**
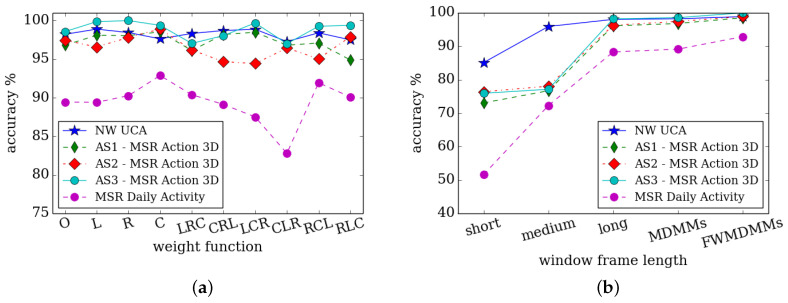
(**a**) Recognition rates based on various weight functions of FWMDMMs concatenated with spatial information. Results are in terms of NUCLA, MSR 3D action subsets and MSR daily activity datasets. The abbreviations, O, L, R and C are the original MDMM, linear, reversed linear and central-oriented fuzzy weight functions, respectively. (**b**) Effects of various lengths of frames based DMMs computation with MDMMs and FWMDMMs on the recognition accuracy.

The MDMMs consist of three lengths of DMMs. These are combined using the linear, reversed linear or centre-oriented weighting fuzzy functions to make up the FWMDMMs. In addition to the above experiments, additional comparisons have been performed. These are based on mixed weighting functions per used length (short, medium and long). These help to show the effects of different weighting functions. This differs from the experiments and implementations that took the same weighting function for the whole three lengths. Figure 11a shows the recognition rates based on various weighting function per length. The abbreviations, *O*, *L*, *R* and *C* refer to the fused spatial information with original MDMMs, linear, reversed linear and centre-oriented FWMDMMs, respectively. The rest of the symbols consist of three letters such as LRC indicate the three weighting functions that used with short, medium and long, respectively. For example, LRC corresponds to DMM computations with: A linear weight function (*L*) for the short length resolution; teversed linear weight function (*R*) with medium length resolution; and centre-oriented weight function (*C*) for the long length resolution. These combine to produce the final FWMDMMs (LRC) and so on for the other combinations.

It can be seen in Figure 11a that using FWMDMM based on different weight functions can significantly improve the recognition rate. It appears that most of the weight functions have helped to achieve better recognition rates in comparison with the original MDMM. For instance, it can be seen that the centre-oriented weight function achieves improved results. This is in terms of MSR daily activity, AS1-MSR action 3D and AS2-MSR action 3D datasets compared to other weight functions and the original MDMM. A reversed linear weight function appears to achieve the best performance in terms of NUCLA and AS3-MSR action 3D datasets. This is in comparison with other weight functions and the original MDMM.

The effect of different lengths (short, medium and long) for the DMM computations can be seen in Figure 11b. This is via the MDMMs and FWMDMMs in terms of the recognition rates for the aforementioned datasets. The HAR rate appears to vary considerably depending on the length of frames that are included in the DMM calculation. Greater lengths appear to result in improved recognition rates. Furthermore, it is noticeable that the different window lengths applied using the fuzzy weight functions also improve the performance. This appears to show that the Fuzzy Multi-resolution formulation encapsulated by FWMDMM might be preferred. This is in comparison to individual windows consisting of short, medium or long and MDMM state models.

## 7. Conclusions

This paper presents the novel FWMDMM for HAR. It appears to utilise the temporal motion information available in depth sequences more effectively compared to traditional DMMs. The feature representation is designed to help provide invariance to variations in action speed. This is important because the same type of action could be performed as different speeds by different people or even the same person. Fuzzy weight functions are employed to help emphasise multiple aspects of an action at different time points. This can help to exploit the most important moments in each action. As a result it can contribute to helping to provide improved differentiation between similar actions. Compact and discriminative features are extracted from FWMDMMs by utilising a CNN based deep motion model. In addition, the spatial and appearance based information in the RGB data and single frame depth data are also utilised. Transfer deep learning from the spatially trained AlexNet CNN is used to effectively represent the spatial information. Different fusion techniques have also been investigated between the spatial and motion information to find the most suitable approach.

The proposed method is able to classify human actions even with small differences in actions. This is in addition to providing excellent performance on actions that partly depend on human–object interactions. The results also appear to show invariance to noisy environments, errors in the depth maps and temporal misalignments.

The proposed approach has been validated on three publicly available benchmark datasets: MSR 3D actions, NUCLA multi-view actions and MSR daily activities. The experiments show that the results from the proposed method are equal if not competitive in comparison to state-of-the-art approaches.

## Figures and Tables

**Table 1:** Recognition accuracy in terms RGB and depth data of the Northwestern-UCLA (NUCLA) dataset using pre-trained AlexNet network.

Validation Scheme	Data	Accuracy %
Cross-Subject	RGB	91
	Depth	88
Cross-view	RGB	20.3
	Depth	21.12

**Table 2:** Recognition accuracy of a KNN classifier in terms of the NUCLA dataset after two AlexNet networks (spatial) fusion.

Settings	Fusion Position	Accuracy %		
		Concatenation	Multiplicative	Addition
Cross-Subject	Conv5	90.49	87.94	91.07
	Max-pooling3	91.20	90.78	89.11
	FC7	94.44	93.88	94.12
	FC8	94.34	93.42	94.18
Cross-view	Conv5	17.67	14.43	15.89
	Max-pooling3	16.87	15.07	15.65
	FC7	24.67	23.54	24.20
	FC8	23.14	22.35	23.07

**Table 3:** A comparison between DMMs and MDMMs concatenated with spatial information (depth and RGB) in terms of the NUCLA dataset.

Settings	DMMs	S+DMMs	MDMMs	S+MDMMs
Cross-subject	93.78	95.83	96.94	98.22
Cross-view	26.13	40.11	53.68	62.94

**Table 4:** Performance of the proposed method in terms of NUCLA dataset using FWMDMMs fused with spatial information (depth and RGB).

Settings	Fusion	S+MDMMs	S+FWMDMMs		
			Linear	Reversed Linear	Centre
Cross-subject	Concatenation	98.22	**98.89**	98.45	97.63
	Multiplicative	96.94	96.83	97.12	95.48
	Addition	97.19	97.10	97.56	96.74
Cross-view	Concatenation	62.94	60.72	66.30	**69.13**
	Multiplicative	49.91	51.42	53.79	49.23
	Addition	52.58	52.17	49.84	52.91

**Table 5:** A comparison between the proposed method and state-of-the-art approaches in terms of the NUCLA dataset.

Paper	Cross-Subject	Cross-View
Virtual view [52]	50.7	47.8
Hankelet [53]	54.2	45.2
MST-AOG [49]	81.6	73.3
Action Bank [54]	24.6	17.6
Poselet [55]	54.9	24.5
tLDS [56]	93.0	74.6
kine-CNN [57]	-	75.6
R-NKTM [58]	-	78.1
Denoised-LSTM [59]	-	79.6
VE-LSTM [60]	-	87.2
E-TS-LSTM [61]	-	89.2
Ours	**98.9**	**69.1**

**Table 6:** Confusion matrix of central-oriented FWMDMMs based method, using cross-view validation scheme in terms of NUCLA dataset.

	Carry	Doffing	Donning	Drop trash	Pick up-one	Pick up-two	Sit down	Stand up	Throw	Walk around
**Carry**	32	0	6	0	0	0	0	0	51	21
**Doffing**	0	88	6	0	0	0	2	0	4	0
**Donning**	0	11	87	0	3	0	0	0	0	0
**Drop trash**	3	11	1	33	14	0	9	6	7	15
**Pick up-one**	0	0	0	0	32	1	33	28	0	5
**Pick up-two**	0	0	0	0	0	100	0	0	0	0
**Sit down**	0	0	0	0	0	0	100	0	0	0
**Stand up**	0	0	0	0	0	0	10	88	0	3
**Throw**	26	8	11	0	9	0	7	4	32	3
**Walk around**	0	0	0	0	0	0	0	0	0	100

**Table 7:** Subsets of MSR action 3D dataset [51].

AS1	AS2	AS3
Horizontal arm wave	High arm wave	High throw
Hammer	Hand catch	Forward kick
Forward punch	Draw tick	Side kick
High throw	Draw cross	Jogging
Hand clap	Draw circle	Tennis swing
Bend	Two-hand wave	Tennis serve
Tennis serve	Side-boxing	Golf swing
Pick-up and throw	Forward kick	Pick-up and throw

**Table 8:** Recognition accuracy in terms depth sequences of MSR 3D action dataset using pre-trained AlexNet network.

Settings	Accuracy %		
	AS1	AS2	AS3
1/3 evaluation scheme	63.99	57.64	65.09
2/3 evaluation scheme	73.48	59.67	68.71
Cross-subject evaluation scheme	45.41	48.61	49.77

**Table 9:** Performance of the deep motion model based on MDMMs and DMMs concatenated with spatial information (depth) in terms of MSR 3D action dataset.

Subsets	Settings	DMMs	S+DMMs	MDMMs	S+MDMMs
AS1	1/3 scheme	74.84	76.71	94.90	**96.81**
	2/3 scheme	82.77	84.13	97.53	**98.72**
	Cross-subjects scheme	50.91	54.35	84.14	**89.78**
AS2	1/3 scheme	76.53	78.07	94.43	**97.44**
	2/3 scheme	77.22	79.30	96.56	**98.11**
	Cross-subjects scheme	62.77	65.15	79.83	**87.61**
AS3	1/3 scheme	76.12	77.15	95.28	**98.57**
	2/3 scheme	81.07	84.79	97.82	**98.97**
	Cross-subjects scheme	58.65	63.85	89.62	**92.15**

**Table 10:** Performance of the proposed method for different weighting functions for the FWMDMMs. Results of different fusion approaches with the spatial information (depth) are also shown. Results are for the MSR 3D action dataset.

Subset	Fusions	Accuracy %								
		Linear			Reverse-Linear			Centre		
		1/3	2/3	CS	1/3	2/3	CS	1/3	2/3	CS
AS1	Concatenate	98.09	98.98	**98.47**	98.05	99.19	93.65	**98.54**	**100**	94.70
	Multiplication	86.95	89.00	82.15	87.34	90.17	79.80	87.66	90.97	81.55
	Addition	90.12	92.87	84.80	87.46	88.20	80.91	89.00	91.17	83.43
AS2	Concatenate	96.58	**100**	94.21	97.81	99.34	91.32	**98.89**	98.49	**96.52**
	Multiplication	85.32	89.04	81.55	88.41	90.29	82.13	91.89	92.80	80.91
	Addition	85.76	93.16	84.70	89.10	91.54	81.43	90.58	89.81	82.30
AS3	Concatenate	99.26	98.57	90.98	**100**	**100**	90.59	99.38	99.84	**94.32**
	Multiplication	83.21	87.90	80.14	84.32	86.56	78.12	82.78	86.22	82.90
	Addition	86.73	89.10	80.59	87.92	90.24	79.61	81.15	88.43	82.63

**Table 11:** Performance of the proposed method compared to the state-of-the-art approaches in terms of the MSR action 3D dataset [51].

Method	Accuracy %											
	1/3 Scheme				2/3 Scheme				Cross Subject Scheme			
	AS1	AS2	AS3	Av.	AS1	AS2	AS3	Av.	AS1	AS2	AS3	Av.
Li et al. [51]	89.5	89.0	96.3	91.6	93.4	92.9	96.3	94.2	71.9	72.9	79.2	74.7
DMM-HOG [17]	97.3	92.2	98.0	95.8	98.7	94.7	98.7	97.4	96.2	84.1	94.6	91.6
Chen et al. [18]	97.3	96.1	98.7	97.4	98.6	98.7	**100**	99.1	96.2	83.2	92.0	90.5
HOJ3D [62]	98.5	96.7	93.5	96.2	98.6	97.2	94.9	97.2	88.0	85.5	63.6	79.0
Evol.Joints [64]	-	-	-	-	-	-	-	-	91.6	90.8	97.3	93.2
DMM-HOG [26]	-	-	-	-	-	-	-	-	90.6	90.7	**99.1**	93.5
Skel.Lie [27]	-	-	-	-	-	-	-	-	95.3	83.9	98.2	92.5
STOP [65]	98.2	94.8	97.4	96.8	99.1	97.0	98.7	98.3	91.7	72.2	98.6	87.5
DMM-LBP-FF [25]	96.7	100	99.3	98.7	100	100	100	100	98.1	92.0	94.6	94.9
DMM-LBP-DF [25]	98.0	97.4	99.3	98.2	100	100	100	100	99.1	92.9	92.0	94.7
WHDMM [66]	-	-	-	-	-	-	-	-	-	-	-	100.0
RNN, LSTM [67]	-	-	-	-	-	-	-	-	-	-	-	94.5
MTL [68]	-	-	-	-	-	-	-	-	-	-	-	95.6
tLDS [56]	-	-	-	-	-	-	-	-	96.8	89.1	98.8	94.9
CNN, SAE [69]	-	-	-	-	-	-	-	-	-	-	-	74.6
3D CNN, DHI [70]	-	-	-	-	-	-	-	-	-	-	-	92.8
VB-DMM [71]	98.0	97.4	99.3	98.2	98.6	100	100	99.5	99.1	92.3	98.2	96.5
DRN [72]	-	-	-	-	-	-	-	-	99.9	99.8	100	99.9
DMLAE [73]	-	-	-	-	-	-	-	-	-	-	-	84.0
**Ours**	**98.5**	**98.8**	**100**	**99.1**	**100**	**100**	**100**	**100**	**98.4**	**96.5**	**94.3**	**96.4**

**Table 12:** Performance of AlexNet network and a KNN classifier based on spatial information (RGB and depth) in terms of MSR daily activity 3D dataset before and after fusion.

Depth	RGB	Fusion Position	Concatenation	Multiplicative	Addition
36	42	Conv5	39.77	34.89	38.17
		Max-pooling3	41.09	40.13	38.74
		FC7	**51.86**	**45.11**	**49.24**
		FC8	51.07	44.21	48.98

**Table 13:** Performance of the deep motion model based on DMMs and MDMMs concatenated with spatial information in terms of MSR daily activity 3D dataset.

Method	Accuracy %
DMMs	67.58
S+DMMs	72.23
MDMMs	85.90
S+MDMMs	**89.14**

**Table 14:** Performance of the proposed method for different weight aspects for the FWMDMMs fused with spatial information (RGB and depth). Results are for the MSR daily activity 3D dataset.

Fusions	Linear	Reversed Linear	Centre-Oriented
Concatenation	89.41	90.26	**92.88**
Multiplication	77.67	79.23	79.52
Addition	79.10	81.29	78.22

**Table 15:** Comparison of our overall proposed method in comparison with state-of-the-art approaches. Results are for the MSR daily activity 3D dataset [50].

Method	Accuracy %
LOP Feature [29]	42.5
STIPs(Harris3D+HOG3D) [74]	60.6
Random Occupancy Pattern [75]	64.0
Joint Position Feature [29]	68.0
STIPs (Cuboids+HOG/HOF) [76]	70.6
Local HON4D [77]	80.0
SNV [51]	86.3
DMMM [62]	81.9
DSTIP+DCSF [30]	83.6
WHDMM [66]	85.0
Actionlet Ensemble [29]	86.0
MDMMs [19]	89.0
CNN, SAE [69]	91.3
MM2DCNN [78]	71.7
MMDT [78]	82.5
Deep Poselets [79]	84.4
DMLAE [73]	67.1
**Ours**	**92.9**

**Table 16:** Confusion matrix of the proposed method based on the concatenation of Centre-oriented FWMDMMs and spatial information in terms of daily activity 3D dataset.

	Drink	Eat	Call cellphone	Use vacuum	Cheer up	Toss paper	Lay down	Walking	Stand up	Sit down
**Drink**	98	2	0	0	0	0	0	0	0	0
**Eat**	3	97	0	0	0	0	0	0	0	0
**Call cellphone**	17	12	70	0	0	0	0	0	0	0
**Use vacuum**	0	0	0	100	0	0	0	0	0	0
**Cheer up**	0	0	0	0	100	0	0	0	0	0
**Toss paper**	10	5	15	0	0	71	0	0	0	0
**Lay down**	0	0	0	0	0	0	100	0	0	0
**Walking**	0	0	0	0	0	0	0	100	0	0
**Stand up**	0	0	0	0	0	0	0	0	98	2
**Sit down**	0	0	0	0	0	0	0	0	6	94

## References

[1] Hasan M., Roy-Chowdhury A.K. (2015). A continuous learning framework for activity recognition using deep hybrid feature models. IEEE Trans. Multimed..

[2] Liu Z., Zhang C., Tian Y. (2016). 3D-based deep convolutional neural network for action recognition with depth sequences. Image Vis. Comput..

[3] Shotton J., Sharp T., Kipman A., Fitzgibbon A., Finocchio M., Blake A., Cook M., Moore R. (2013). Real-time human pose recognition in parts from single depth images. Commun. ACM.

[4] Yang X., Tian Y. Super normal vector for activity recognition using depth sequences. Proceedings of the IEEE Conference on Computer Vision and Pattern Recognition.

[5] Hinton G.E., Osindero S., Teh Y.W. (2006). A fast learning algorithm for deep belief nets. Neural Comput..

[6] LeCun Y., Bottou L., Bengio Y., Haffner P. (1998). Gradient-based learning applied to document recognition. Proc. IEEE.

[7] Sun K., Zhang J., Zhang C., Hu J. (2017). Generalized extreme learning machine autoencoder and a new deep neural network. Neurocomputing.

[8] Hochreiter S., Schmidhuber J. (1997). Long short-term memory. Neural Comput..

[9] Le Q.V., Zou W.Y., Yeung S.Y., Ng A.Y. Learning hierarchical invariant spatio-temporal features for action recognition with independent subspace analysis. Proceedings of the 2011 IEEE Conference on Computer Vision and Pattern Recognition (CVPR).

[10] Wang J., Chen Y., Hao S., Peng X., Hu L. (2018). Deep learning for sensor-based activity recognition: A Survey. Pattern Recognit. Lett..

[11] Rawat W., Wang Z. (2017). Deep convolutional neural networks for Image Classification: A Comprehensive Review. Neural Comput..

[12] Zhang Z., Geiger J., Pohjalainen J., Mousa A.E.D., Jin W., Schuller B. (2018). Deep learning for environmentally robust speech recognition: An overview of recent developments. ACM Trans. Intell. Syst. Technol. (TIST).

[13] Goyal S., Benjamin P. (2014). Object recognition using deep neural networks: A survey. arXiv.

[14] Sun L., Jia K., Chan T.H., Fang Y., Wang G., Yan S. DL-SFA: Deeply-learned slow feature analysis for action recognition. Proceedings of the IEEE Conference on Computer Vision and Pattern Recognition.

[15] Wang P., Li W., Gao Z., Zhang J., Tang C., Ogunbona P. (2015). Deep convolutional neural networks for action recognition using depth map sequences. arXiv.

[16] Wang P., Li W., Ogunbona P., Wan J., Escalera S. (2018). RGB-D-based human motion recognition with deep learning: A survey. Comput. Vis. Image Underst..

[17] Yang X., Zhang C., Tian Y. (2012). Recognizing actions using depth motion maps-based histograms of oriented gradients. Proceedings of the 20th ACM international conference on Multimedia.

[18] Chen C., Liu K., Kehtarnavaz N. (2016). Real-time human action recognition based on depth motion maps. J. Real Time Image Process..

[19] Chen C., Liu M., Liu H., Zhang B., Han J., Kehtarnavaz N. (2017). Multi-Temporal depth motion maps-Based local binary patterns for 3-D human action recognition. IEEE Access.

[20] Chen C., Liu M., Zhang B., Han J., Jiang J., Liu H. 3D Action Recognition Using Multi-Temporal depth motion maps and Fisher Vector. Proceedings of the Twenty-Fifth International Joint Conference on Artificial Intelligence (IJCAI ’16).

[21] Zhang Z. (2012). Microsoft kinect sensor and its effect. IEEE Multimed..

[22] Haggag H., Hossny M., Filippidis D., Creighton D., Nahavandi S., Puri V. Measuring depth accuracy in RGB-D cameras. Proceedings of the 2013 7th International Conference on Signal Processing and Communication Systems (ICSPCS).

[23] Ali H.H., Moftah H.M., Youssif A.A. (2017). Depth-based human activity recognition: A comparative perspective study on feature extraction. Future Comput. Inform. J..

[24] Ye M., Zhang Q., Wang L., Zhu J., Yang R., Gall J. (2013). A Survey on Human Motion Analysis from Depth Data. Time-of-Flight and Depth Imaging. Sensors, Algorithms, and Applications.

[25] Chen C., Jafari R., Kehtarnavaz N. Action recognition from depth sequences using depth motion maps-based local binary patterns. Proceedings of the 2015 IEEE Winter Conference on Applications of Computer Vision (WACV).

[26] El Madany N.E.D., He Y., Guan L. human action recognition using temporal hierarchical pyramid of depth motion map and keca. Proceedings of the 2015 IEEE 17th International Workshop on Multimedia Signal Processing (MMSP).

[27] Vemulapalli R., Arrate F., Chellappa R. human action recognition by representing 3D skeletons as points in a lie group. Proceedings of the IEEE Conference on Computer Vision and Pattern Recognition.

[28] Zhu Y., Chen W., Guo G. (2014). Evaluating spatiotemporal interest point features for depth-based action recognition. Image Vis. Comput..

[29] Wang J., Liu Z., Wu Y. (2014). Learning Actionlet Ensemble for 3D human action recognition. Human Action Recognition with Depth Cameras.

[30] Xia L., Aggarwal J. Spatio-temporal depth cuboid similarity feature for activity recognition using depth camera. Proceedings of the 2013 IEEE Conference on Computer Vision and Pattern Recognition (CVPR).

[31] Yang X., Tian Y. (2017). Super normal vector for human activity recognition with depth cameras. IEEE Trans. Pattern Anal. Mach. Intell..

[32] Wang X., Gao L., Song J., Zhen X., Sebe N., Shen H.T. (2018). Deep appearance and motion learning for egocentric activity recognition. Neurocomputing.

[33] Vella F., Augello A., Maniscalco U., Bentivenga V., Gaglio S. Classification of Indoor Actions through Deep Neural Networks. Proceedings of the 2016 12th International Conference on Signal-Image Technology & Internet-Based Systems (SITIS).

[34] Gowda S.N. Human activity recognition using combinatorial Deep Belief Networks. Proceedings of the 2017 IEEE Conference on Computer Vision and Pattern Recognition Workshops (CVPRW).

[35] Ji S., Xu W., Yang M., Yu K. (2013). 3D convolutional neural networks for human action recognition. IEEE Trans. Pattern Anal. Mach. Intell..

[36] Wu H., Gu X. (2015). Max-pooling dropout for regularization of convolutional neural networks. Proceedings of the International Conference on Neural Information Processing.

[37] Yu K., Xu W., Gong Y. (2009). Deep Learning with Kernel Regularization for Visual Recognition. Advances in Neural Information Processing Systems.

[38] Han Y., Zhang P., Zhuo T., Huang W., Zhang Y. (2018). Going deeper with two-stream ConvNets for action recognition in video surveillance. Pattern Recognit. Lett..

[39] Sun L., Jia K., Yeung D.Y., Shi B.E. human action recognition using factorized spatio-temporal convolutional networks. Proceedings of the IEEE International Conference on Computer Vision.

[40] Simonyan K., Zisserman A. (2014). Two-Stream Convolutional Networks for Action Recognition in Videos. Advances in Neural Information Processing Systems.

[41] Wang L., Qiao Y., Tang X. Action recognition with trajectory-pooled deep-convolutional descriptors. Proceedings of the IEEE Conference on Computer Vision and Pattern Recognition.

[42] Park E., Han X., Berg T.L., Berg A.C. Combining multiple sources of knowledge in deep cnns for action recognition. Proceedings of the 2016 IEEE Winter Conference on Applications of Computer Vision (WACV).

[43] Feichtenhofer C., Pinz A., Wildes R.P. Spatiotemporal multiplier networks for video action recognition. Proceedings of the 2017 IEEE Conference on Computer Vision and Pattern Recognition (CVPR).

[44] Feichtenhofer C., Pinz A., Zisserman A. Convolutional Two-Stream Network Fusion for Video Action Recognition. Proceedings of the 2016 IEEE Conference on Computer Vision and Pattern Recognition (CVPR).

[45] Karpathy A., Toderici G., Shetty S., Leung T., Sukthankar R., Fei-Fei L. Large-scale video classification with convolutional neural networks. Proceedings of the IEEE conference on Computer Vision and Pattern Recognition.

[46] Chen C., Jafari R., Kehtarnavaz N. (2016). A real-time human action recognition system using depth and inertial sensor fusion. IEEE Sens. J..

[47] Krizhevsky A., Sutskever I., Hinton G.E. (2012). Imagenet Classification with Deep convolutional neural networks. Advances in Neural Information Processing Systems.

[48] Thaker D., Krishnakumar K. (2017). k-Shot Learning for Action Recognition. https://pdfs.semanticscholar.org/7576/8ff4129ca6cd122c5ca729e9cfc66cc798fe.pdf.

[49] Wang J., Nie X., Xia Y., Wu Y., Zhu S.C. Cross-view action modeling, learning and recognition. Proceedings of the IEEE Conference on Computer Vision and Pattern Recognition.

[50] Wang J., Liu Z., Wu Y., Yuan J. Mining actionlet ensemble for action recognition with depth cameras. Proceedings of the 2012 IEEE Conference on Computer Vision and Pattern Recognition (CVPR).

[51] Li W., Zhang Z., Liu Z. Action recognition based on a bag of 3D points. Proceedings of the 2010 IEEE Computer Society Conference on Computer Vision and Pattern Recognition Workshops (CVPRW).

[52] Li R., Zickler T. Discriminative virtual views for cross-view action recognition. Proceedings of the 2012 IEEE Conference on Computer Vision and Pattern Recognition (CVPR).

[53] Li B., Camps O.I., Sznaier M. Cross-view activity recognition using hankelets. Proceedings of the 2012 IEEE Conference on Computer Vision and Pattern Recognition (CVPR).

[54] Sadanand S., Corso J.J. Action bank: A high-level representation of activity in video. Proceedings of the 2012 IEEE Conference on Computer Vision and Pattern Recognition (CVPR).

[55] Maji S., Bourdev L., Malik J. Action recognition from a distributed representation of pose and appearance. Proceedings of the 2011 IEEE Conference on Computer Vision and Pattern Recognition (CVPR).

[56] Ding W., Liu K., Belyaev E., Cheng F. (2018). Tensor-based linear dynamical systems for action recognition from 3D skeletons. Pattern Recognit..

[57] Wang J., Liu Y. Kinematics Features for 3D Action Recognition Using Two-Stream CNN. Proceedings of the 2018 13th World Congress on Intelligent Control and Automation (WCICA).

[58] Rahmani H., Mian A., Shah M. (2017). Learning a deep model for human action recognition from novel viewpoints. IEEE Trans. Pattern Anal. Mach. Intell..

[59] Demisse G., Papadopoulos K., Aouada D., Ottersten B. Pose encoding for robust skeleton-based action recognition. Proceedings of the Conference on Computer Vision and Pattern Recognition Workshops.

[60] Baptista R., Ghorbel E., Papadopoulos K., Demisse G.G., Aouada D., Ottersten B. View-invariant Action Recognition from RGB Data via 3D Pose Estimation. Proceedings of the 2019 IEEE International Conference on Acoustics, Speech and Signal Processing (ICASSP 2019).

[61] Lee I., Kim D., Kang S., Lee S. Ensemble deep learning for skeleton-based action recognition using temporal sliding lstm networks. Proceedings of the IEEE International Conference on Computer Vision.

[62] Xia L., Chen C.C., Aggarwal J. View invariant human action recognition using histograms of 3D joints. Proceedings of the 2012 IEEE Computer Society Conference on Computer Vision and Pattern Recognition Workshops (CVPRW).

[63] Padilla-López J.R., Chaaraoui A.A., Flórez-Revuelta F. (2014). A discussion on the validation tests employed to compare human action recognition methods using the MSR action 3D dataset. arXiv.

[64] Chaaraoui A.A., Padilla-López J.R., Climent-Pérez P., Flórez-Revuelta F. (2014). Evolutionary joint selection to improve human action recognition with RGB-D devices. Expert Syst. Appl..

[65] Vieira A.W., Nascimento E.R., Oliveira G.L., Liu Z., Campos M.F. (2014). On the improvement of human action recognition from depth map sequences using space–time occupancy patterns. Pattern Recognit. Lett..

[66] Wang P., Li W., Gao Z., Zhang J., Tang C., Ogunbona P. (2016). Action recognition from depth maps using deep convolutional neural networks. IEEE Trans. Hum. Mach. Syst..

[67] Du Y., Wang W., Wang L. Hierarchical recurrent neural network for skeleton based action recognition. Proceedings of the IEEE Conference on Computer Vision and Pattern Recognition.

[68] Yang Y., Deng C., Tao D., Zhang S., Liu W., Gao X. (2016). Latent max-margin multitask learning with skelets for 3-D action recognition. IEEE Trans. Cybern..

[69] Tomas A., Biswas K. Human activity recognition using combined deep architectures. Proceedings of the 2017 IEEE 2nd International Conference on Signal and Image Processing (ICSIP).

[70] Keçeli A.S., Kaya A., Can A.B. (2018). Combining 2D and 3D deep models for action recognition with depth information. Signal Image Video Process..

[71] Jin K., Jiang M., Kong J., Huo H., Wang X. (2017). Action recognition using vague division DMMs. J. Eng..

[72] Pham H.H., Khoudour L., Crouzil A., Zegers P., Velastin S.A. (2018). Exploiting deep residual networks for human action recognition from skeletal data. Comput. Vis. Image Underst..

[73] Yin X., Chen Q. Deep metric learning autoencoder for nonlinear temporal alignment of human motion. Proceedings of the 2016 IEEE International Conference on Robotics and Automation (ICRA).

[74] Klaser A., Marszałek M., Schmid C. (2008). A spatio-temporal descriptor based on 3D-gradients. Proceedings of the 19th British Machine Vision Conference.

[75] Wang J., Liu Z., Chorowski J., Chen Z., Wu Y. (2012). Robust 3D Action Recognition with Random Occupancy Patterns. Computer Vision—ECCV 2012.

[76] Dollár P., Rabaud V., Cottrell G., Belongie S. Behavior recognition via sparse spatio-temporal features. Proceedings of the 2nd Joint IEEE International Workshop on Visual Surveillance and Performance Evaluation of Tracking and Surveillance.

[77] Oreifej O., Liu Z. Hon4d: Histogram of oriented 4d normals for activity recognition from depth sequences. Proceedings of the IEEE Conference on Computer Vision and Pattern Recognition.

[78] Asadi-Aghbolaghi M., Bertiche H., Roig V., Kasaei S., Escalera S. Action recognition from RGB-D data: Comparison and fusion of spatio-temporal handcrafted features and deep strategies. Proceedings of the IEEE International Conference on Computer Vision.

[79] Mavroudi E., Tao L., Vidal R. Deep moving poselets for video based action recognition. Proceedings of the 2017 IEEE Winter Conference on Applications of Computer Vision (WACV).

